# Auxin controls rice root angle via kinase OsILA1-mediated cell wall modifications

**DOI:** 10.1126/sciadv.ady2320

**Published:** 2025-09-19

**Authors:** Xiaoyun Song, Suhang Yu, Qiaoyi Li, Yali Xiong, Dilixiadanmu Tashenmaimaiti, Malcolm J. Bennett, Staffan Persson, Xiuzhen Kong, Rahul Bhosale, Guoqiang Huang

**Affiliations:** ^1^ Joint International Research Laboratory of Metabolic & Developmental Sciences, School of Life Sciences and Biotechnology, Shanghai Jiao Tong University, Shanghai, China.; ^2^ School of Biosciences, University of Nottingham, Nottingham LE12 5RD, UK.; ^3^ Department of Plant & Environmental Sciences (PLEN), Copenhagen Plant Science Center (CPSC), University of Copenhagen, Frederiksberg 1870, Denmark.

## Abstract

Auxin-mediated root gravitropism critically determines root angle, a key trait that underpins root system architecture (RSA). Auxin response factors (ARFs) regulate this process, but their downstream targets and functions remain unclear. We demonstrate that OsARF12 and OsARF25 induce cell wall thickness in epidermal cells of the lower side of the root, positioning auxin as a “break” of these cells during gravistimulation by directly regulating *Increased Leaf Angle1* (*OsILA1*) through a dual set of auxin response elements. Our study therefore establishes a direct link between auxin transcriptional signaling and cell wall fortification, driving the differential cell elongation required for root gravitropism and angle.

## Teaser

Auxin-driven transcriptional reprogramming alters cell wall thickness, shaping rice root gravitropism.

## INTRODUCTION

Root angle, a crucial characteristic of the root system—an important interface mediating plant-environment interactions, is predominantly shaped by root gravitropism, which occurs in three distinct phases: gravity perception, asymmetric auxin distribution, and differential growth response (*1*–*5*). The differential growth is governed by an asymmetric auxin gradient that forms within the root elongation zone, leading to differential cell elongation (*6*, *7*). According to the acid growth theory, low auxin concentrations on the upper side induce cell expansion through cell wall acidification and relaxation (*8*). In contrast, high auxin accumulation on the lower side of the gravitropic root inhibits cell elongation (*9*). Further research has shown that auxin orchestrates two opposing signaling cascades, rapidly modulating the apoplastic pH to influence cell wall expansion (*10*, *11*). A transmembrane kinase, TRANSMEMBRANE KINASE1 (TMK1), interacts with plasma membrane H^+^-ATPase and mediates its phosphorylation to promote extracellular acidification; meanwhile, canonical auxin signaling within the cells drives net cellular H^+^ influx, resulting in extracellular alkalization (*10*). The simultaneous activation of these antagonistic mechanisms enables roots to precisely modulate differential cell elongation between the upper and lower sides of the gravitropic root.

Auxin signaling operates through the degradation of AUXIN/INDOLE-3-ACETIC ACID (Aux/IAA) proteins and is mediated by active auxin response factor (ARF) proteins (*12*, *13*). Active ARFs (AtARF7 and AtARF19), the pivotal transcriptional regulators of auxin signaling, play crucial roles in the root gravitropic response in *Arabidopsis* (*14*). However, the downstream effectors of the active ARFs and the intricate regulatory mechanisms governing root gravitropic response remain elusive (*15*). In this study, we identify two rice active ARFs, OsARF12 and OsARF25, which directly activate *OsILA1* expression at the lower epidermis of gravitropic roots. This up-regulation enhances the thickness of the epidermal cell wall on the lower side of gravitropic roots, thereby inhibiting cell elongation and promoting gravitropic bending.

## RESULTS

### Root gravitropic responses are impaired in *OsILA1* mutants

**Fig. 1.**
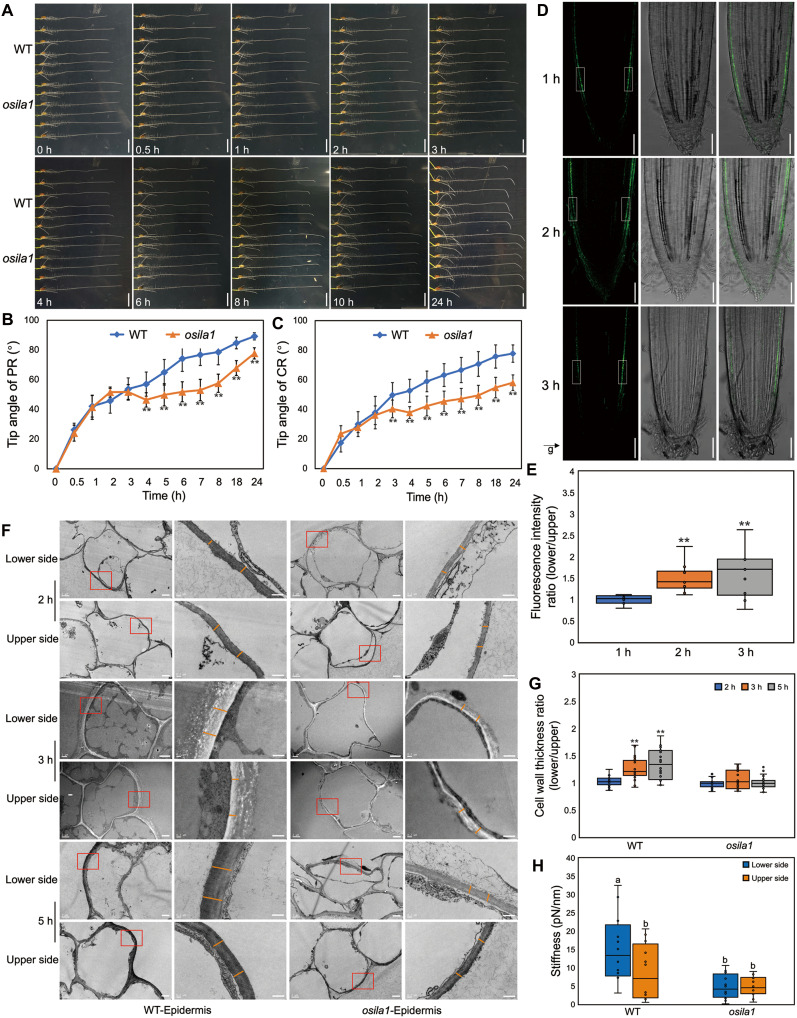
*OsILA1* mutant exhibits reduced gravitropic response. (**A**) Representative root images of WT and *osila1* with 24-hour gravitropic responses. Scale bars, 1 cm. h, ours. (**B** and **C**) Tip angle analysis of primary root (B) and crown root (C) of WT and *osila1*. Error bars are ± SD, *n* = 12. Student’s *t* test: **P < 0.01. (**D**) Representative confocal images, bright-field images, and merge images of the root longitudinal section of the rice *proOsILA1::OsILA1-NG*/*osila1* (*OsILA1-NG*) translational reporter after 1- to 3-hour gravitational stimulation. Scale bars, 100 μm. (**E**) Fluorescence intensity ratio (lower side/upper side) analysis of the rice *OsILA1-NG* translational reporter in primary root after 1- to 3-hour gravitational stimulation. Box plots in (E) showing the intensity ratio of fluorescence signals in the region of interest (ROI) of the lower side (right) versus upper side (left) of elongation zone. Error bars are ± SD, *n* = 10. Student’s *t* test: **P < 0.01. (**F**) Representative cell wall images of the upper side and lower side of the WT and *osila1* root epidermis after 2- to 5-hour gravitational stimulation. The left pictures of WT and *osila1* are epidermis cells. Scale bars, 2 μm. The magnified view on the right is the region marked by the red box. The orange line indicates the cell wall. Scale bars, 0.5 μm. (**G**) Cell wall thickness ratio of the lower side versus the upper side of the root epidermis. Error bars are ± SD, *n* ≥ 40 positions. Different letters indicate significant differences, *P* < 0.01 from using a one-way ANOVA. (**H**) Force spectroscopy results showing stiffness values between WT and *osila1*. Error bars are ± SD, *n* ≥ 10 positions. Different letters indicate significant differences, *P* < 0.05 from a one-way ANOVA.

OsILA1, a Raf-like mitogen-activated protein kinase kinase kinase (MAPKKK) of group C with serine/threonine kinase activity, plays a pivotal role in cell wall biosynthesis and controls leaf angle (*16*–*18*). We found that *OsILA1* was specifically expressed in the rice root epidermis (fig. S1), which led us to hypothesize its function in root growth and development. Following gravistimulation, both primary and crown roots of *OsILA1* mutant seedlings exhibited weaker gravitropism compared to wild-type (WT) plants (Fig. 1, A to C). Temporal analysis revealed that *osila1* exhibited a weaker gravitropic response (and normal root elongation rate) compared to WT only after 3 hours of gravistimulation (Fig. 1, A to C, and fig. S2A). This delayed response suggests that OsILA1 acts during the root bending growth response, following gravity sensing and auxin gradient formation. To avoid the impact of using transplanted seedlings on the experiment, we conducted gravitropic assays with nontransplanted seedlings and obtained consistent results (fig. S2B). Notably, following reorientation, differential expression of *OsILA1* was observed between the lower and upper epidermis as early as 2 hours after gravistimulation, highlighting the link between gravitropism and the expression pattern of *OsILA1* (Fig. 1, D and E).

OsILA1 is essential for cell wall biosynthesis, which is critical for plant cell morphology (*16*). To investigate whether OsILA1 influences cell wall production during root gravitropism, we prepared ultrathin cross sections of the gravitropic response zone and examined them using transmission electron microscopy (TEM). Because *OsILA1* is expressed in epidermal cells, we measured the thickness of root epidermal cell walls in both WT and *osila1* mutant roots (fig. S3A). No significant differences were observed in the epidermal cell wall thickness between the upper and lower sides at the nonbending site or the upper side of the bending site in WT roots (fig. S3B), whereas the lower side exhibited increased cell wall thickness after 3-hour gravistimulation (fig. S3B), suggesting that the lower side may play a predominant role in gravitropic responses. Consistently, in WT plants, the ratio of epidermal cell wall thickness (lower side/upper side) at the bend showed no significant difference after 2-hour gravistimulation but significantly increased after 3-hour gravistimulation (Fig. 1, F and G). In contrast, the *osila1* mutant displayed no significant difference in cell wall thickness between the two sides (Fig. 1, F and G), suggesting that OsILA1 primarily functions in the high auxin signaling pathway at the lower side of gravitropic roots. Intriguingly, thinner cell wall and normal elongation rate were monitored in *osila1* root (figs. S2A and S3B), suggesting distinct regulatory pathways governing root angle and root length. As anticipated, the stiffness of the cell wall was greater at the epidermis on the lower side versus the upper side of WT gravitropic roots, whereas no significant differences were observed in *osila1* plants (Fig. 1H). These findings highlight the critical role of increased cell wall thickness regulation in the lower side of root gravitropism and demonstrate that OsILA1 regulates this process.

### Auxin regulates root gravitropism via OsILA1

Root gravitropic responses in *Arabidopsis thaliana* are attributed to the asymmetric distribution and response of auxin in the epidermis (*9*). Similarly, we observed a comparable pattern of auxin response in the epidermis of elongating root tissues in rice (fig. S4A). Notably, the asymmetric fluorescence signal (detected after 1 hour of gravistimulation) appeared earlier than the corresponding *OsILA1* expression signal (observed after 2 hours of gravistimulation) (Fig. 1E and fig. S4B). This temporal relationship suggests that OsILA1 may function downstream of auxin signaling to regulate root gravitropism.

To further explore the relationship between auxin signaling and *OsILA1* in root gravitropism, we analyzed *OsILA1* expression in WT roots treated with two concentrations of naphthaleneacetic acid (NAA) (10 and 100 nM), an auxin analog. Our results showed that *OsILA1* expression in rice roots was significantly induced only at higher concentrations of NAA (100 nM) (fig. S5, A and B). Notably, the roots of WT and *osila1* elongated at similar rates at lower auxin concentrations (10 nM; fig. S5, C and D). However, root elongation in WT was inhibited at 100 nM NAA, whereas the *osila1* mutant showed no such inhibition (fig. S5, C and D). Consistent with these findings, WT roots exhibited thicker cell walls under high auxin treatment (100 nM), whereas *osila1* mutants did not (fig. S5, E and F). Notably, this response was absent under low auxin conditions (10 nM) (fig. S5, E and F). These results demonstrate that OsILA1 regulates cell wall fortification in response to high, but not low, auxin concentrations.

**Fig. 2.**
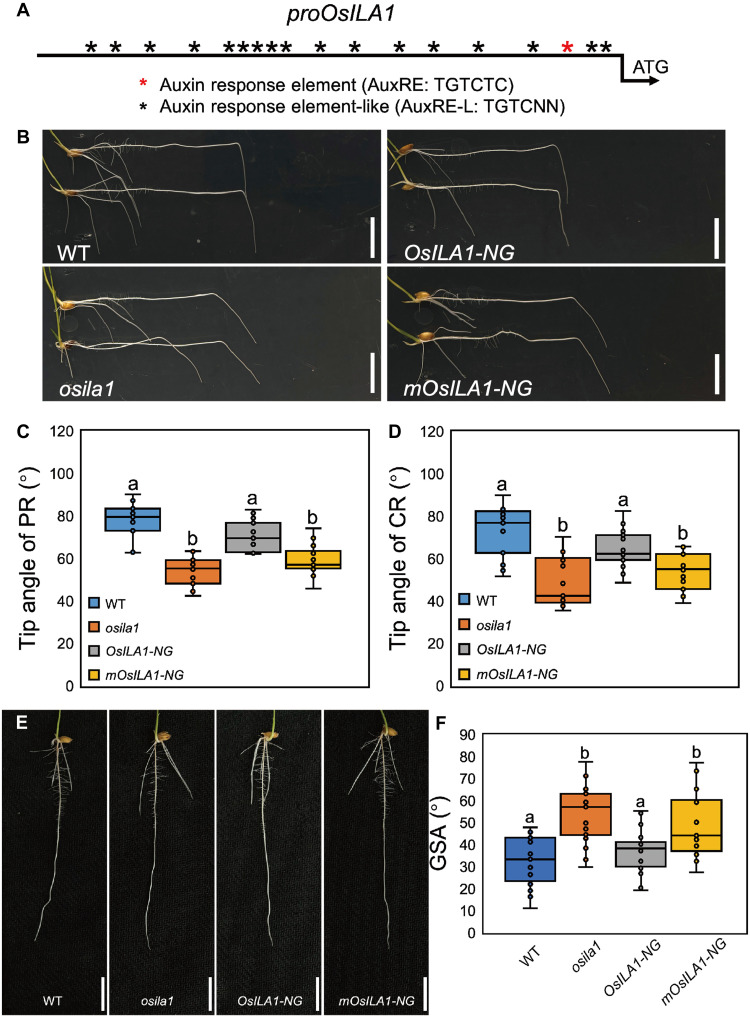
AuxRE element is essential for the expression of *OsILA1*. (**A**) Diagram of the *OsILA1* promoter region. Red asterisks: AuxRE (TGTCTC); black asterisks: AuxRE-L (TGTCNN). (**B**) Representative root images of WT, *osila1*, *proOsILA1::OsILA1-NG/osila1* (*OsILA1-NG*), and *mproOsILA1::OsILA1-NG/osila1* (*mOsILA1-NG*) with 12-hour gravitropic responses. Scale bars, 1 cm. (**C** and **D**) Tip angle analysis of PR (C) and CR (D) of WT, *osila1*, *OsILA1-NG*, and *mOsILA1-NG*. Error bars are ± SD, *n* = 15. Different letters indicate significant differences, *P* < 0.01 from a one-way ANOVA. (**E**) Representative root systems of WT, *osila1*, *OsILA1-NG*, and *mOsILA1-NG* with 7-day growth. Scale bars, 1 cm. (**F**) GSA analysis of WT, *osila1*, *OsILA1-NG*, and *mOsILA1-NG*. Error bars are ± SD, *n* = 10. Different letters indicate significant differences, *P* < 0.01 from a one-way ANOVA.

Given that *OsILA1* is regulated by auxin, we next scanned the promoter region of the gene for any auxin-related cis-elements. The *OsILA1* promoter contains a canonical auxin response element (AuxRE) as well as multiple noncanonical AuxREs (Fig. 2A), suggesting that *OsILA1* expression may be directly regulated by auxin-mediated transcriptional signaling, potentially involving OsARFs. To determine the importance of these cis-elements in *OsILA1* regulation, we complemented the *osila1* mutant with *OsILA1* cDNA driven by either a native or mutated AuxRE (i.e., TGTCTC to TGGCTC) *OsILA1* promoter. The *osila1* mutant, characterized by reduced gravitropism (Fig. 2, B to D) and a shallower root system (Fig. 2, E and F), was fully rescued by the native *OsILA1* promoter construct, whereas transgenic lines with the mutated promoter failed to rescue the *osila1* mutant phenotypes (Fig. 2, B to F). Consistently, transgenic plants with mutated AuxRE promoter constructs displayed relative insensitivity to auxin treatment, i.e., reduced induction of *OsILA1* by auxin, compared to the elevated *OsILA1* expression in lines with the native *OsILA1* promoter (fig. S6). These results suggest that auxin induces *OsILA1* expression via AuxRE elements in the root epidermis and that this induction is important for rice root gravitropism and root angle determination.

### OsARF12 and OsARF25 regulate root gravitropism

**Fig. 3.**
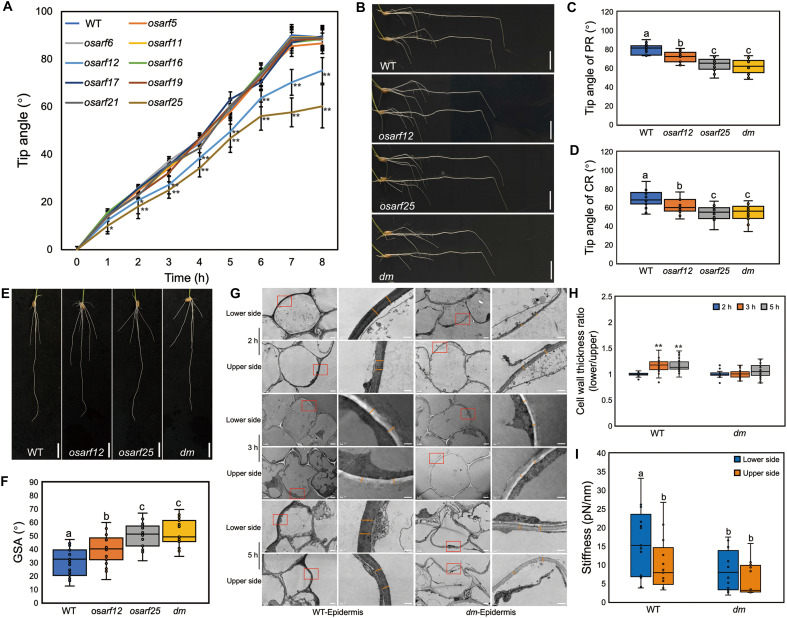
*OsARF12* and *OsARF25* mutants exhibit shallow root systems. (**A**) Tip angle analysis of PR of WT and *osarf5/6/11/12/16/17/19/21/25* with 8-hour gravitropic responses. Error bars are ± SD, *n* = 12. Student’s *t* test: **P < 0.01. (**B**) Representative root images of gravitropic responses in WT, *osarf12*, *osarf25*, and *osarf12 osarf25* double mutant (*dm*). Scale bars, 1 cm. (**C**) Tip angle analysis of WT, *osarf12*, *osarf25*, and *dm* PR after 12-hour gravitropic responses. Error bars are ± SD, *n* = 15. Different letters indicate significant differences, *P* < 0.01 from a one-way ANOVA. (**D**) Tip angle analysis of WT, *osarf12*, *osarf25*, and *dm* CR after 12-hour gravitropic responses. Error bars are ± SD, *n* = 25. Different letters indicate significant differences, *P* < 0.01 from a one-way ANOVA. (**E**) Representative root systems of WT, *osarf12*, *osarf25*, and *dm* with 7-day growth. Scale bars, 1 cm. (**F**) GSA analysis of WT, *osarf12*, *osarf25*, and *dm*. Error bars are ± SD, *n* = 10. Different letters indicate significant differences, *P* < 0.01 from a one-way ANOVA. (**G**) Representative cell wall images of WT and *dm* root epidermis (upper/lower sides) after gravistimulation. Left: Epidermal cells (scale bars, 2 μm). Right: Magnified red box regions with cell walls marked (orange lines; scale bars, 0.5 μm). (**H**) Thickness ratio of the lower side versus the upper side of the root epidermis cell wall. Error bars are ± SD, *n* ≥ 40 positions. Different letters indicate significant differences, *P* < 0.01 from a one-way ANOVA. (**I**) Force spectroscopy results showing stiffness values between WT and *dm*. Error bars are ± SD, *n* ≥ 10 positions. Different letters indicate significant differences, *P* < 0.05 from a one-way ANOVA.

AuxRE elements are typically regulated by ARFs (*19*). To genetically investigate potential roles of OsARFs in *OsILA1*-mediated root gravitropism, we mutated all nine activator *OsARFs* in the rice genome, specifically *OsARF5*, *OsARF6*, *OsARF11*, *OsARF12*, *OsARF16*, *OsARF17*, *OsARF19*, *OsARF21*, and *OsARF25* (figs. S7 to S14). Of these, only the *osarf12* or *osarf25* mutants exhibited an attenuated root gravitropic response (Fig. 3A and figs. S7 to S14). Transcriptional reporter analysis revealed that both *OsARF12* and *OsARF25* are highly expressed not only in roots, including the epidermis, but also in the ectoderm, cortex, endoderm, and stele (fig. S15). A previous study in *Arabidopsis* showed that AtARF7 and AtARF19 regulate root gravitropism redundantly (*14*). To examine the relationship of the closely related rice homologs (*20*, *21*), we crossed the *osarf12* and *osarf25* single mutants to generate a double mutant (hereafter termed *dm*). Phenotypic characterization of both primary and crown roots in *dm* lines revealed a similar phenotype to that of the *osarf25* single mutant (Fig. 3, B to D). Consistently, similar to single *osarf25* mutant, *dm* exhibited a shallower root system compared to WT plants (Fig. 3, E and F). These results suggested that both OsARF12 and OsARF25 have roles in root gravitropism but that OsARF25 may play a more dominant role.

To determine whether OsARF12 and OsARF25 are involved in regulating differential cell wall biosynthesis during gravitropism, similar to OsILA1, we measured the thickness of the epidermal cell wall of WT and *dm* using TEM during gravitropic response. Consistent with that of the *osila1* mutant, no significant difference in cell wall thickness of epidermal cells was observed between the two sides in the *dm* mutant in comparison with the induced cell wall thickness at the lower side of WT gravitropic root from 3-hour gravistimulation (Fig. 3, G and H). No significant differences were observed in the epidermal cell wall thickness between the upper and lower sides at both the bending site and nonbending site in *dm* roots (fig. S16). In addition, thinner cell walls were detected in *dm* root epidermis (fig. S16). We also observed comparable results to the *osila1* mutant regarding the stiffness of the cell wall (Fig. 3I). These results indicate that OsARF12 and OsARF25 are involved in the regulation of differential cell wall biosynthesis that drives root gravitropism.

### OsARF12 and OsARF25 directly activate *OsILA1* expression

**Fig. 4.**
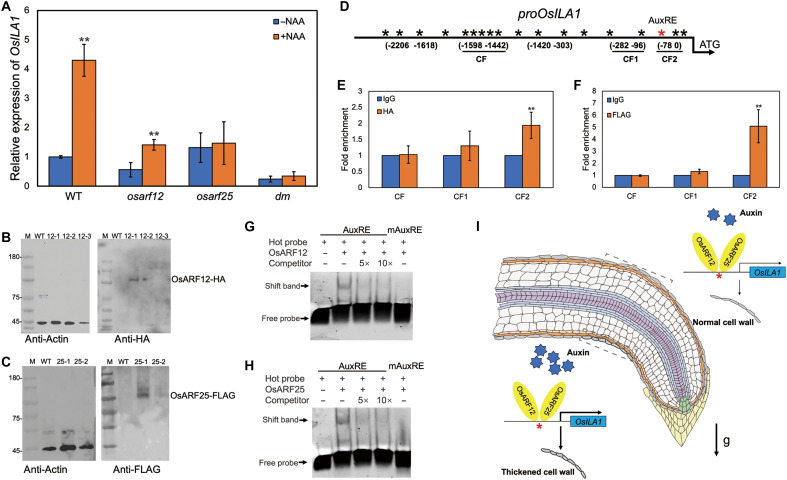
OsARF12 and OsARF25 directly activate *OsILA1* expression. (**A**) Relative expression of *OsILA1* in response to 100 nM NAA treatment in WT, *osarf12*, *osarf25*, and *dm*, as determined by RT-qPCR. Error bars indicate ± SE from three biological replicates. Student’s *t* test: **P < 0.01. (**B**) Abundance of OsARF12 in OsARF12 transgenic plants. (**C**) Abundance of OsARF25 in OsARF25 transgenic plants. (**D**) Diagram of the *OsILA1* promoter region. Red asterisks: AuxRE (TGTCTC); black asterisks: AuxRE-L (TGTCNN). (**E**) ChIP-qPCR assays to detect interaction in roots between OsARF12 and CF1/2. CF1 fragment contains three AuxRE-Ls; CF2 comprises one AuxRE and two Aux RE-Ls. Error bars indicate ± SE from three biological replicates. Student’s *t* test: **P < 0.01. IgG, immunoglobulin G. (**F**) ChIP-qPCR assays to detect interaction in roots between OsARF25 and CF1/2. Error bars indicate ± SE from three biological replicates. Student’s *t* test: **P < 0.01. (**G** and **H**) EMSA showing the direct binding of OsARF12/OsARF25 to AuxRE motif of CF2 probes of the *OsILA1* promoter. A 5- and 10-fold excess of nonlabeled probes was used for competitive binding assays. The labeled probes with mutated AuxRE site were used for control. (**I**) Proposed model for this study. High auxin accumulates in the lower epidermis of the root after gravitational stimulation. OsARF12 and OsARF25 directly bind to the canonical AuxRE of the *OsILA1* promoter to activate its higher epidermis expression. OsILA1 controls root gravitropism via up-regulating cell wall biosynthesis to reduce the growth of lower side epidermal cells, resulting in a larger root angle.

Given the phenotypic similarities among *osila1*, *osarf12*, and *osarf25* mutants during gravitropism, we first assessed the regulatory relationship between OsARF12 and OsARF25 and *OsILA1* expression using reverse transcription quantitative polymerase chain reaction (RT-qPCR). Our results showed that *OsILA1* expression was reduced in both *osarf12* and *osarf25* single mutants and further reduced in the *dm* lines (Fig. 4A). Whereas *OsILA1* expression was significantly up-regulated by the NAA treatment in the WT (Fig. 4A), such induction was suppressed in the *osarf12* mutant (Fig. 4A). Moreover, *OsILA1* expression was not induced by NAA in either the *osarf25* or *dm* lines (Fig. 4A), suggesting that *OsILA1* expression is dependent on *OsARF12* and *OsARF25*, with *OsARF25* playing a particularly critical role.

To confirm the binding affinity of OsARF12 and OsARF25 to the promoter region of *OsILA1*, we generated transgenic rice plants expressing OsARF12 and OsARF25 fused with hemagglutinin (HA) and FLAG tags, respectively (Fig. 4, B and C). Chromatin immunoprecipitation–quantitative polymerase chain reaction (ChIP-qPCR) analysis revealed significant enrichment of the CF2 promoter fragment (which contains the canonical AuxRE and 2 AuxRE-like motifs) for both OsARF12 and OsARF25 (Fig. 4, D to F). In addition, electrophoretic mobility shift assay (EMSA) demonstrated a clear gel shift in the probes containing the AuxRE site rather than the mutated AuxRE site when incubated with recombinant OsARF12 and OsARF25 proteins (Fig. 4, G and H), confirming their ability to bind directly to the AuxRE site of the *OsILA1* promoter.

Overall, we outline a mechanism for how auxin regulates cell growth during the root gravitropic response. OsARF12 and OsARF25 directly induce *OsILA1* expression in the epidermal cells on the lower side of the gravitropic root, promoting cell wall biosynthesis, resulting in a thicker cell wall and attenuated cell elongation (Fig. 4I).

## DISCUSSION

The suppression of root growth in response to elevated auxin levels underpins gravitropic bending response and facilitates land plants’ anchorage in soil (*9*). The rapid effects of auxin on root growth have been clarified through the identification of contrasting mechanisms: cellular TMK-based apoplast acidification and intracellular canonical auxin signaling–induced apoplast alkalinization (*10*, *15*), whereas the “slow” regulatory mechanisms by which canonical auxin signaling inhibits root growth remain inadequately understood (*10*). In this study, we demonstrate that two rice ARFs (OsARF12 and OsARF25) directly activate the expression of *OsILA1* in the epidermis on the lower side of gravitropic roots, and this activation leads to increased cell wall thickness, thereby inhibiting cell elongation and promoting gravitropic bending (Fig. 4I). Nevertheless, other signaling pathways independent of the OsARF12/25-OsILA1 cascade may also contribute to the regulation of root gravitropism.

Our findings contribute to the ongoing debate regarding the rapid versus long-term, cellular versus transcriptional mechanism of auxin signaling in the inhibition of root elongation. Our results indicate that canonical ARF-based transcriptional regulation occurs subsequent to nontranscriptional signaling in an irreversible manner. Hence, it is plausible that OsARFs–OsILA1–cell wall cascade further reinforces the rapid inhibitory effects of elevated auxin level on root elongation. This OsARF12/25-mediated regulatory process provides a crucial component in the auxin signaling pathway. Notably, *OsARF12* and/or *OsARF25* mutants display reduced gravitropic response earlier than *osila1* plants (Figs. 1B and 3A), implying that additional interacting partners or downstream regulators associated with OsARF12 and OsARF25 are likely to play a crucial role in the early stages of the gravitropic response.

Root angle is a key agronomic trait of root system that determines a plant’s access to water in deep soil layers or phosphate resources in surface soil layers. This study provides insight into the transcriptional regulation of auxin-mediated cell growth and offers a potential pathway for manipulating root growth angles to cultivate new crop lines with enhanced adaptation to soil environments.

## MATERIALS AND METHODS

### Plant materials and growth conditions

All genetic backgrounds of rice cultivars generated in this study were produced in the Zhonghua11 (ZH11) (*Oryza sativa* L. ssp. *Japonica*) background. The *osarf12 osarf25* double mutant was obtained by self-crossing F_2_ populations from homozygous *osarf12* and *osarf25* plants. Rice plants were cultured in Shanghai (30°N, 121°E) in the summer and in Sanya (18°N, 109°E), China in the winter. The genetic transformation of rice was performed by EDGENEBIOT Company (https://www.edgene.com.cn/).

The seeds were germinated in the dark for 4 days. For nontransplanted gravitropism, the germinated seeds were transferred on the plates with 1% agar for 5 days and then were placed horizontally. For transplanted gravitropism, the germinated seeds were transferred in the black box with water for 5 days. The 5-day seedlings of plates were transferred on 1% agar for 2 hours and then placed horizontally. The root angle was measured from images using ImageJ software (National Institutes of Health, Bethesda, MD, USA). The gravitropic setpoint angle (GSA) of crown roots was measured at the insertion site between the crown root (CR) and primary root (PR) concerning the gravity vector. For the gravitropism analysis of crown roots at different angles, the seeds were germinated for 3 days and grown in paper bags for 7 days. For auxin treatment, rice seeds were germinated in water under dark conditions for 4 days. The germinated seeds were transferred on the PCR plates without bottom and grown for 4 days. To determine the auxin sensitivity of ZH11 and *osila1*, the seedlings were treated with either 10 or 100 nM NAA for 5 days.

### Plasmid construction

All cloned PCR amplifications were done with KOD One PCR Master Mix (TOYOBO) with the suggested annealing temperature and extension time according to fragment length. To create stable *OsARF12* and *OsARF25* transgenic rice plants, the coding sequence (CDS) of *OsARF12* was cloned into the *pCAMBIA1301* vector with the 3 X HA tag, and the CDS of *OsARF25* was cloned into the *pEXT06/g* vector with the 3 X FLAG tag. The positive plants were screened using hygromycin B (hyg). To create stable *proOsARF12::VENUS-N7*, *proOsARF25::VENUS-N7*, and *proOsILA1::VENUS-N7* transgenic rice plants, ~2-kb promoters upstream of the start codon were cloned into the *pCAMBIA1301* vector with the *VENUS-N7* reporter gene. The fluorescence was excited by 514-nm laser and recorded between 525 and 550 nm for VENUS-N7. The fluorescence of the reconstituted VENUS-N7 protein was visualized using confocal laser scanning microscopy with a TCS SP5 laser scanning confocal microscope. The promoter and CDS were cloned into the *pCAMBIA3301* vector with the *mNeonGreen* reporter gene to generate *proOsILA1::OsILA1-NG* and *mproOsILA1::OsILA1-NG* with mutated AuxRE (TGTCTC to TGGCTC), which were then introduced into Agrobacterium strain EHA105 and transformed into the *osila1* mutant to generate the complement plants. The fluorescence was excited by 488-nm laser and recorded between 500 and 550 nm for mNeonGreen. All the primers used for the vector constructions in this study are listed in table S1.

### Reverse transcription quantitative polymerase chain reaction

An RT-qPCR analysis was performed using a LightCycler 480 Real-Time PCR system (Roche). WT, *osarf12*, *osarf25*, and *osarf12 osarf25* seeds were germinated for 4 days. The germinated seeds were transferred to the black box and grown in water for 4 days then treated with 100 nM NAA for 2 hours. The 5-mm root tips of the seedlings were frozen in liquid nitrogen, and total RNA was extracted from 100 mg of frozen seedlings using the RNAprep Pure Tissue Kit (Tiangen Biotech, Beijing, China), and first-strand cDNA was synthesized using a cDNA Synthesis Kit (Takara Bio, Kusatsu, Japan). The relative expression values were determined using *TUB2* as the reference gene and plotted relative to its expression level in WT. WT seeds were grown in water for 4 days, and then the germinated seeds were transferred on the PCR plates without bottom for 5 days and then treated with either 10 or 100 nM NAA for 2 hours. The 5-mm root tips were harvested for studies. The related primers used for qPCR are listed in table S1.

### Western blot analysis

Total proteins were isolated from the 5-mm root tips of the 5-day seedlings, and 20 μg of protein from each sample was loaded onto a 10% gel for SDS–polyacrylamide gel electrophoresis. After running for 2 hours, the proteins were in situ electrotransferred onto polyvinyl difluoride (PVDF) membranes (Merck, Darmstadt, Germany). The membranes were incubated in phosphate-buffered saline (PBS) containing 5% bovine serum albumin (BSA) and 0.05% Tween 20 for 60 min, washed three times, and then probed with a monoclonal antibody overnight at 4°C. Proteins and epitope-tagged proteins were detected using Pierce ECL Western Blotting Substrate (Thermo Fisher Scientific).

### ChIP-qPCR assay

Roots (5-day seedlings) were cross-linked in 1% (w/v) formaldehyde under vacuum for 15 min, quenched by 0.25 M glycine for 8 min, washed five times in ddH_2_O, and ground in liquid nitrogen. Nuclear proteins were precipitated by centrifugation at 2880*g* and sheared into 100– to 300–base pair (bp) fragments by sonication; nonsonicated chromatin DNA was reverse cross-linked and used as the total input DNA control. IP was performed using anti-HA and anti-FLAG antibodies (mouse monoclonal, M20003 and M20008, Abmart) bound to protein G agarose/salmon sperm DNA (16-201, Sigma-Aldrich). The IP proteins and DNA were eluted with 1% (w/v) SDS and 0.1 M NaHCO_3_, and the cross-linking was reversed by incubation in 250 mM NaCl at 65°C for 12 hours. qPCR was performed on IP genomic DNA, and the relative enrichment was normalized to input for each sample (primers in table S1).

### Electrophoretic mobility shift assay

The full-length CDS of *OsARF12* and *OsARF25* were amplified and cloned into pGADT7 (Clontech, TaKaRa, Shiga, Japan) for protein expression by an in vitro translation kit (TNT T7/SP6 Coupled Wheat Germ Extract System; Promega, Madison, WI, USA). Double-stranded AuxRE hot probe was synthesized with forward/reverse primers with and without 5′-end modification with 6-carboxyfluorescein (6-FAM) dye. The competitors were the double-stranded oligonucleotide without any modification. The control group is the double-stranded oligonucleotide with mutated AuxRE (TGTCTC to TGGCTC). The binding reaction mixture [binding buffer: 250 mM tris-acetate, 10 mM dithiothreitol, BSA (1 mg/ml), and 20 mM MgAC_2_] was incubated at 25°C for 20 min. Competition assays were tested using 5- and 10-fold nonlabeled probes. EMSA reaction products were run in a 6% polyacrylamide gel.

### Clearing procedure

Rice roots were fixed with 4% paraformaldehyde under vacuum for 1 hour. After being rinsed three times in 1× PBS, the plant material was transferred to the ClearSee solution (10% xylitol, 15% sodium deoxycholate, and 25% urea) and cleared at room temperature. For rice young roots (4 days old), 3-day clearing is sufficient. The ClearSee solution was replaced several times during the period.

### TEM analysis

The seeds were germinated in the dark for 4 days. After culturing the seeds of WT, *osila1*, and *dm* in the water for 5 days, gravistimulation treatments were applied for 2, 3, and 5 hours, respectively. Subsequently, materials from the bending sites of root tips were collected for each group and fixed in 2.5% glutaraldehyde under vacuum. Two milliliters of 0.1 M phosphate buffer (PB) composed of 0.2 M NaH_2_PO_4_ and 0.2 M Na_2_HPO_4_ was added, and the material was rinsed three times, each for 15 min. Following this, the material was fixed with 1% osmium acid on a shaker for 2 to 3 hours. The material was rinsed three times with 2 ml of 0.1 M PB, each for 15 min. Subsequently, gradient dehydration was performed on a shaker using 20, 40, 60, 70, 90, and 100% ethanol, each concentration for 30 min. After replacing the ethanol with propylene oxide, the material was infiltrated with resin. Last, the infiltrated material was embedded in a mold. After embedding, ultrathin sections of 100 nm were cut with an ultramicrotome (EM UC6-FC6, Leica). After staining with uranium acetate and lead citrate, sections were imaged using TEM (HT7650, Hitachi) at 80 kV.

### Atomic force microscopy analysis of rice root cell walls

Five-day-old seedlings were transplanted onto 1% agar plates and subjected to vertical positioning in the dark for 2 hours at 28°C. To induce gravitropic stimulation, the plates were carefully rotated 90° for 3 hours and 50-μm longitudinal sections of the gravitropic response zone were prepared and examined using atomic force microscopy (Dimension IconXR, Bruker). The ratio of force (nN) to distance (nm) was measured to characterize the rigidity of the cell wall.

### Statistical analysis

The statistical significance of any difference between two groups was determined using Student’s *t* test, whereas the statistical significance of differences between multiple groups was determined using a one-way analysis of variance (ANOVA).

## References

[1] L. R. Band, D. M. Wells, A. Larrieu, J. Y. Sun, A. M. Middleton, A. P. French, G. Brunoud, E. M. Sato, M. H. Wilson, B. Peret, M. Oliva, R. Swarup, I. Sairanen, G. Parry, K. Ljung, T. Beeckman, J. M. Garibaldi, M. Estelle, M. R. Owen, K. Vissenberg, T. C. Hodgman, T. P. Pridmore, J. R. King, T. Vernoux, M. J. Bennett, Root gravitropism is regulated by a transient lateral auxin gradient controlled by a tipping-point mechanism. Proc. Natl. Acad. Sci. U.S.A. 109, 4668–4673 (2012). 22393022 10.1073/pnas.1201498109 PMC3311388

[2] L. Abas, R. Benjamins, N. Malenica, T. Paciorek, J. Wisniewska, J. C. Moulinier-Anzola, T. Sieberer, J. Friml, C. Luschnig, Intracellular trafficking and proteolysis of the Arabidopsis auxin-efflux facilitator PIN2 are involved in root gravitropism. Nat. Cell Biol. 8, 249–256 (2006). 16489343 10.1038/ncb1369

[3] G. Huang, W. Liang, C. J. Sturrock, B. K. Pandey, J. Giri, S. Mairhofer, D. Wang, L. Muller, H. Tan, L. M. York, J. Yang, Y. Song, Y.-J. Kim, Y. Qiao, J. Xu, S. Kepinski, M. J. Bennett, D. Zhang, Rice actin binding protein RMD controls crown root angle in response to external phosphate. Nat. Commun. 9, 2346 (2018). 29892032 10.1038/s41467-018-04710-x PMC5995806

[4] J. Giri, R. Bhosale, G. Q. Huang, B. K. Pandey, H. Parker, S. Zappala, J. Yang, A. Dievart, C. Bureau, K. Ljung, A. Price, T. Rose, A. Larrieu, S. Mairhofer, C. J. Sturrock, P. White, L. Dupuy, M. Hawkesford, C. Perin, W. Q. Liang, B. Peret, C. T. Hodgman, J. Lynch, M. Wissuwa, D. B. Zhang, T. Pridmore, S. J. Mooney, E. Guiderdoni, R. Swarup, M. J. Bennett, Rice auxin influx carrier *OsAUX1* facilitates root hair elongation in response to low external phosphate. Nat. Commun. 9, 1408 (2018). 29650967 10.1038/s41467-018-03850-4 PMC5897452

[5] G. K. Kirschner, F. Hochholdinger, S. Salvi, M. J. Bennett, G. Q. Huang, R. A. Bhosale, Genetic regulation of the root angle in cereals. Trends Plant Sci. 29, 814–822 (2024). 38402016 10.1016/j.tplants.2024.01.008

[6] J. Friml, X. Yang, M. Michniewicz, D. Weijers, A. Quint, O. Tietz, R. Benjamins, P. B. Ouwerkerk, K. Ljung, G. Sandberg, P. J. Hooykaas, K. Palme, R. Offringa, A PINOID-dependent binary switch in apical-basal PIN polar targeting directs auxin efflux. Science 306, 862–865 (2004). 15514156 10.1126/science.1100618

[7] J. Kleine-Vehn, Z. J. Ding, A. R. Jones, M. Tasaka, M. T. Morita, J. Friml, Gravity-induced PIN transcytosis for polarization of auxin fluxes in gravity-sensing root cells. Proc. Natl. Acad. Sci. U.S.A. 107, 22344–22349 (2010). 21135243 10.1073/pnas.1013145107 PMC3009804

[8] D. L. Rayle, R. Cleland, Enhancement of wall loosening and elongation by acid solutions. Plant Physiol. 46, 250–253 (1970). 16657445 10.1104/pp.46.2.250 PMC396573

[9] R. Swarup, E. M. Kramer, P. Perry, K. Knox, H. M. O. Leyser, J. Haseloff, G. T. S. Beemster, R. Bhalerao, M. J. Bennett, Root gravitropism requires lateral root cap and epidermal cells for transport and response to a mobile auxin signal. Nat. Cell Biol. 7, 1057–1065 (2005). 16244669 10.1038/ncb1316

[10] L. X. Li, I. Verstraeten, M. Roosjen, K. Takahashi, L. Rodriguez, J. Merrin, J. Chen, L. Shabala, W. Smet, H. Ren, S. Vanneste, S. Shabala, B. De Rybel, D. Weijers, T. Kinoshita, W. M. Gray, J. Friml, Cell surface and intracellular auxin signalling for H^+^ fluxes in root growth. Nature 599, 273–277 (2021). 34707283 10.1038/s41586-021-04037-6 PMC7612300

[11] W. W. Lin, X. Zhou, W. X. Tang, K. Takahashi, X. Pan, J. W. Dai, H. Ren, X. Y. Zhu, S. Q. Pan, H. Y. Zheng, W. M. Gray, T. D. Xu, T. Kinoshita, Z. B. Yang, TMK-based cell-surface auxin signalling activates cell-wall acidification. Nature 599, 278–282 (2021). 34707287 10.1038/s41586-021-03976-4 PMC8549421

[12] S. Kepinski, O. Leyser, Ubiquitination and auxin signaling: A degrading story. Plant Cell 14, S81–S95 (2002). 12045271 10.1105/tpc.010447 PMC151249

[13] F. D. Maraschin, J. Memelink, R. Offringa, Auxin-induced, SCFTIR1-mediated poly-ubiquitination marks AUX/IAA proteins for degradation. Plant J. 59, 100–109 (2009). 19309453 10.1111/j.1365-313X.2009.03854.x

[14] Y. Okushima, P. J. Overvoorde, K. Arima, J. M. Alonso, A. Chan, C. Chang, J. R. Ecker, B. Hughes, A. Lui, D. Nguyen, C. Onodera, H. Quach, A. Smith, G. X. Yu, A. Theologis, Functional genomic analysis of the AUXIN RESPONSE FACTOR gene family members in Arabidopsis thaliana: Unique and overlapping functions of ARF7 and ARF19. Plant Cell 17, 444–463 (2005). 15659631 10.1105/tpc.104.028316 PMC548818

[15] M. Fendrych, M. Akhmanova, J. Merrin, M. Glanc, S. Hagihara, K. Takahashi, N. Uchida, K. U. Torii, J. Friml, Rapid and reversible root growth inhibition by TIR1 auxin signalling. Nat Plants 4, 453–459 (2018). 29942048 10.1038/s41477-018-0190-1 PMC6104345

[16] J. Ning, B. C. Zhang, N. L. Wang, Y. H. Zhou, L. Z. Xiong, Increased leaf angle1, a Raf-like MAPKKK that interacts with a nuclear protein family, regulates mechanical tissue formation in the lamina joint of rice. Plant Cell 23, 4334–4347 (2011). 22207574 10.1105/tpc.111.093419 PMC3269869

[17] D. M. Zhang, Z. P. Xu, S. X. Cao, K. L. Chen, S. C. Li, X. L. Liu, C. X. Gao, B. C. Zhang, Y. H. Zhou, An uncanonical CCCH-tandem zinc-finger protein represses secondary wall synthesis and controls mechanical strength in rice. Mol. Plant 11, 163–174 (2018). 29175437 10.1016/j.molp.2017.11.004

[18] G. Q. Huang, H. Hu, A. van de Meene, J. Zhang, L. Dong, S. Zheng, F. L. Zhang, N. S. Betts, W. Q. Liang, M. J. Bennett, S. Persson, D. B. Zhang, AUXIN RESPONSE FACTORS 6 and 17 control the flag leaf angle in rice by regulating secondary cell wall biosynthesis of lamina joints. Plant Cell 33, 3120–3133 (2021). 34245297 10.1093/plcell/koab175 PMC8462825

[19] T. J. Guilfoyle, G. Hagen, Auxin response factors. Curr. Opin. Plant Biol. 10, 453–460 (2007). 17900969 10.1016/j.pbi.2007.08.014

[20] Y. H. Qi, S. K. Wang, C. J. Shen, S. N. Zhang, Y. Chen, Y. X. Xu, Y. Liu, Y. R. Wu, D. A. Jiang, OsARF12, a transcription activator on auxin response gene, regulates root elongation and affects iron accumulation in rice (Oryza sativa). New Phytol. 193, 109–120 (2012). 21973088 10.1111/j.1469-8137.2011.03910.x

[21] D. K. Wang, K. M. Pei, Y. P. Fu, Z. X. Sun, S. J. Li, H. Q. Liu, K. Tang, B. Han, Y. Z. Tao, Genome-wide analysis of the auxin response factors (ARF) gene family in rice (Oryza sativa). Gene 394, 13–24 (2007). 17408882 10.1016/j.gene.2007.01.006

