# Supplementary material for: Auxin controls rice root angle via kinase OsILA1-mediated cell wall modifications
Source: Sci Adv. 2025 Sep 19;11(38):eady2320. doi: 10.1126/sciadv.ady2320 (PMC12448151; doi:10.1126/sciadv.ady2320)
Supplement: Supplementary file 1 — Figs. S1 to S16 Table S1 [file sciadv.ady2320_sm.pdf]

Science Advances
AAAS

Supplementary Materials for

Auxin controls rice root angle via kinase OsILA1-mediated cell wall modifications

Xiaoyun Song *et al.*

Corresponding author: Guoqiang Huang, huang19880901@sjtu.edu.cn;
Rahul Bhosale, rahul.bhosale@nottingham.ac.uk; Xiuzhen Kong, xzkong1988@sjtu.edu.cn

Sci. Adv. **11**, eady2320 (2025)
DOI: 10.1126/sciadv.ady2320

This PDF file includes:

Figs. S1 to S16
Table S1

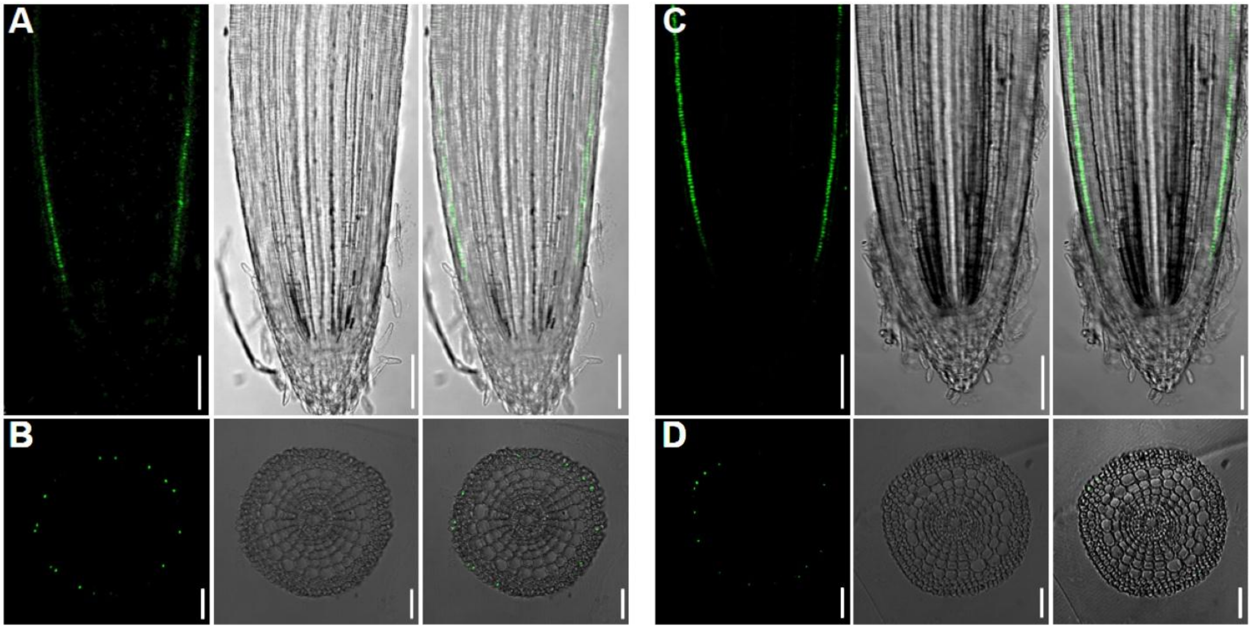

Fig. S1. OsILA1 was accumulated in epidermis. (**A**) Representative confocal images, bright field images and merge images of the root longitudinal section of rice *proOsILA1::VENUS-N7* transcriptional reporter. Scale bar, 100 µm. (**B**) Representative confocal images, bright field images and merge images of the root transverse section of rice *proOsILA1::VENUS-N7* transcriptional reporter. Scale bar, 100 µm. (**C**) Representative confocal images, bright field images and merge images of the root longitudinal section of rice *proOsILA1::OsILA1-NG/osila1* translational reporter. Scale bar, 100 µm. (**D**) Representative confocal images, bright field images and merge images of the root transverse section of rice *proOsILA1::OsILA1-NG/osila1* translational reporter. Scale bar, 100 µm.

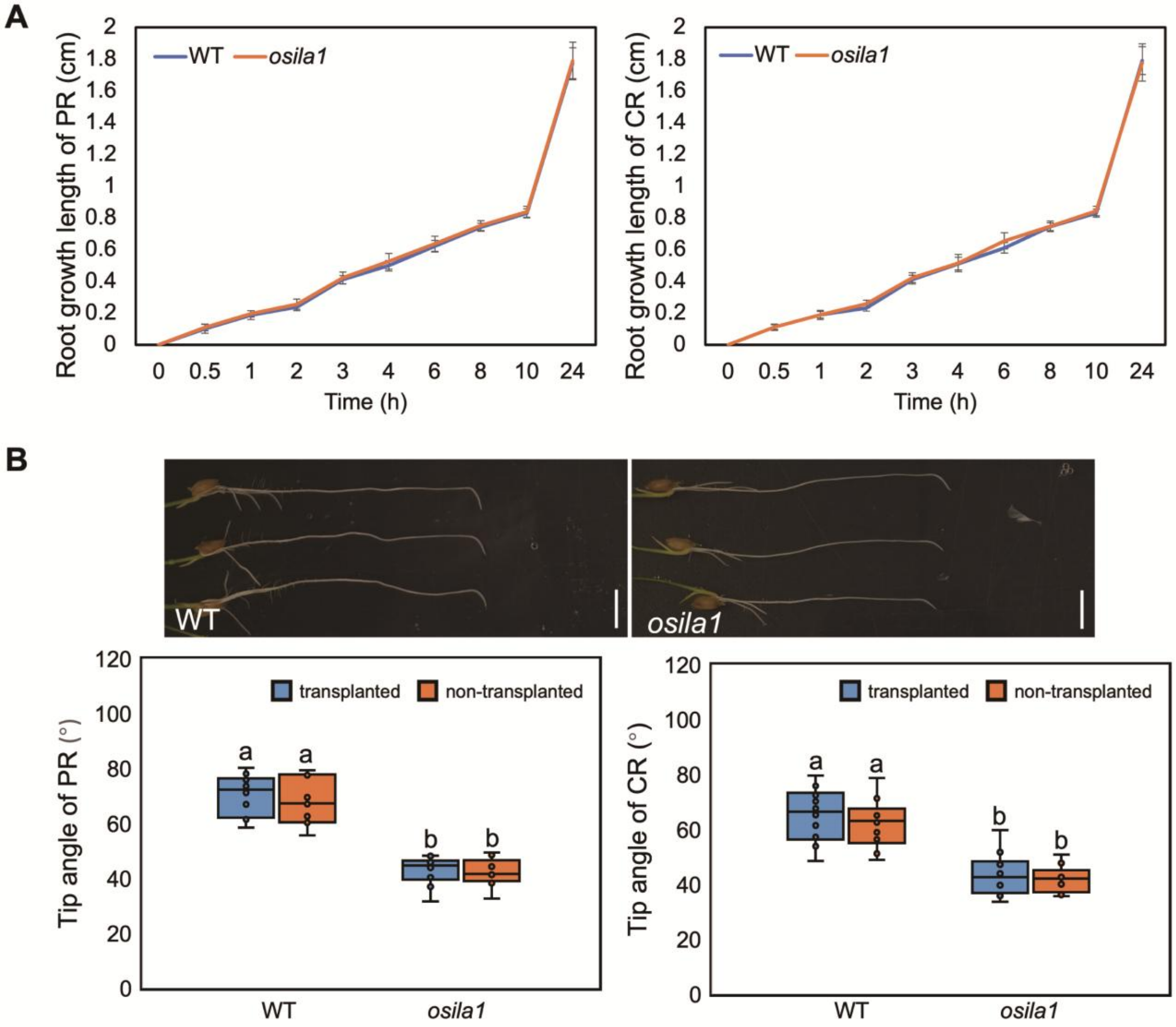

Fig. S2. ***OsILA1*** **mutants exhibit reduced gravitropic response.** (**A**) Primary root (PR) and crown root (CR) growth length analysis of WT and *osila1* after gravitropic responses. (**B**) Representative root images and tip angle analysis after 12-h gravitropic responses in transplanted and non-transplanted seedlings of WT and *osila1*. Scale bars, 1 cm. Error bars are ± SD, n = 15. Different letters indicate significant differences, $P < 0.01$ from a one-way ANOVA.

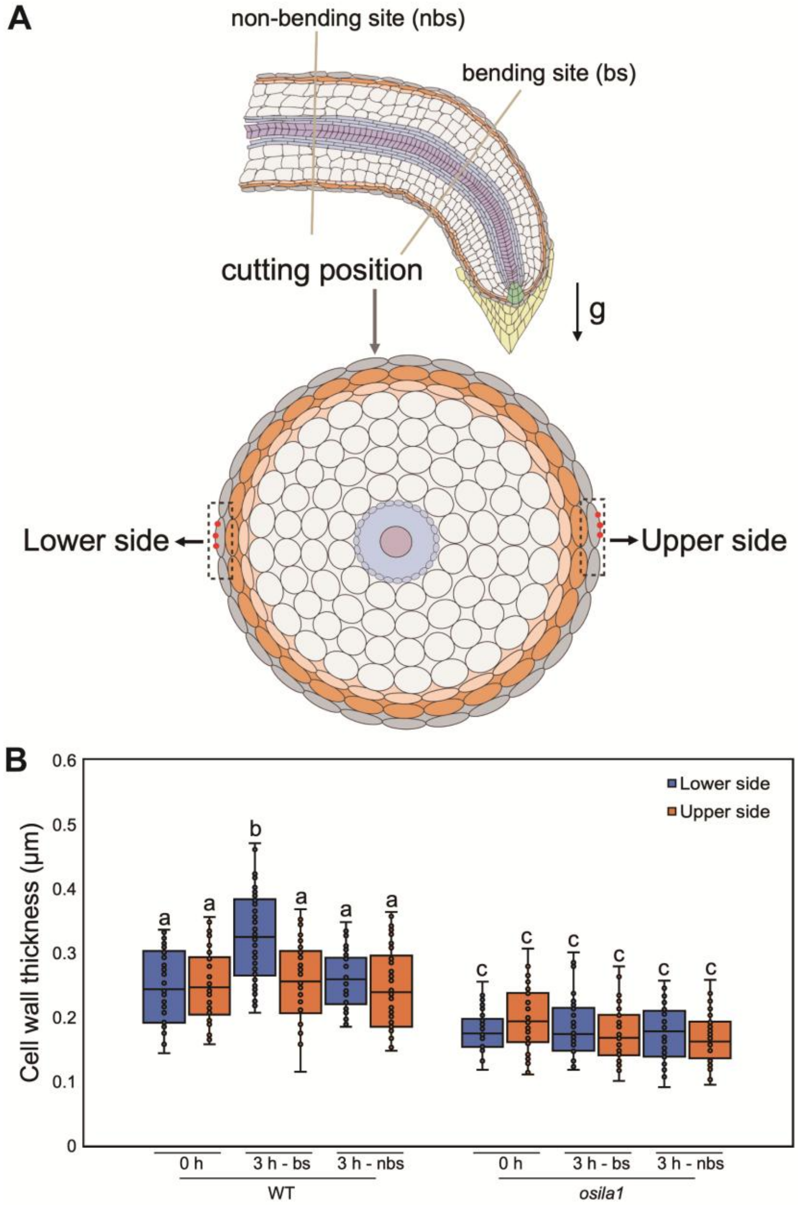

Fig. S3. *OsILA1* mutants show thinner cell wall thickness. (**A**) Schematic diagram of ultrathin sections. 5-day-old seedlings were transplanted onto 1% agar plates and subjected to vertical positioning in the dark for 1 h. The plates were carefully rotated 90° for 3 hrs and ultrathin cross-sections of the gravitropic response zone were prepared and examined using TEM. When we trim the resin block, mark the lower side as serrated and the upper side as circular. The thickness of three points (red dots as indicated in the figure) on the lower and upper sides of the epidermal cells was measured. (**B**) The cell wall thickness of the upper and lower sides of the root epidermis at 0 and after 3-h of gravistimulation. Error bars are ± SD, n ≥ 30 positions. Different letters indicate significant differences, $P < 0.01$ from using a one-way ANOVA.

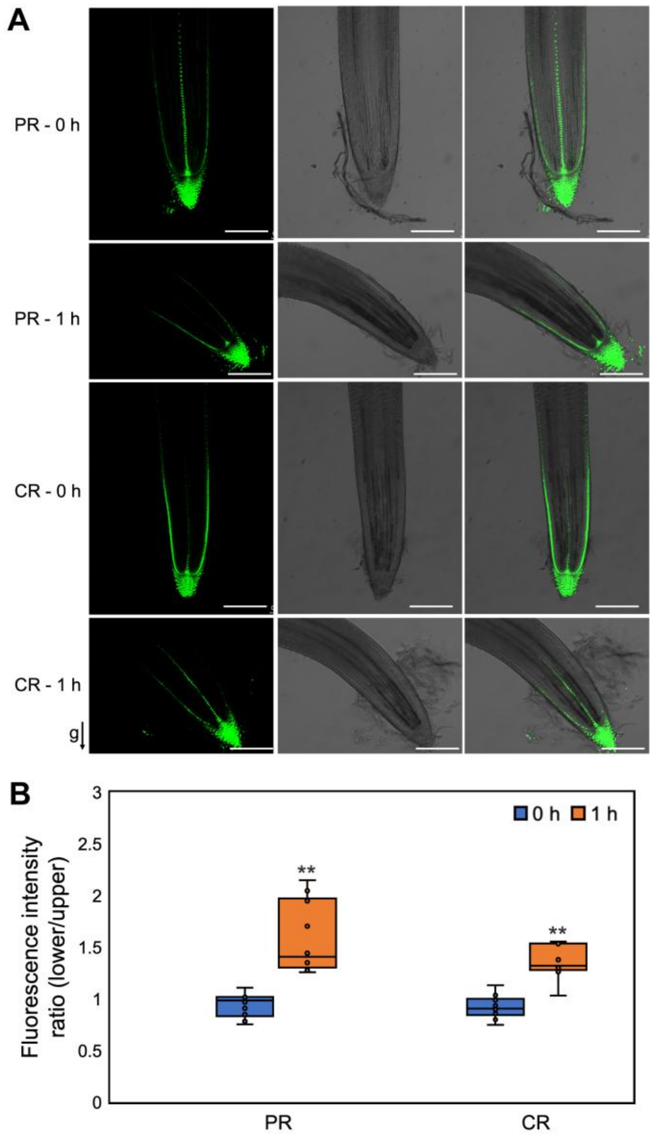

Fig. S4. Auxin response dynamics in rice root. (**A**) Representative confocal images of the primary root (PR) and crown root (CR) longitudinal sections of rice *DR5-VENUS* at 0 and after 1-h of gravistimulation. Scale bar, 250 µm. (**B**) Intensity ratio of fluorescence signals of the lower side versus upper side of elongation zone. Error bars are ± SD, n = 10. Student's *t*-test: $^{**}P < 0.01$.

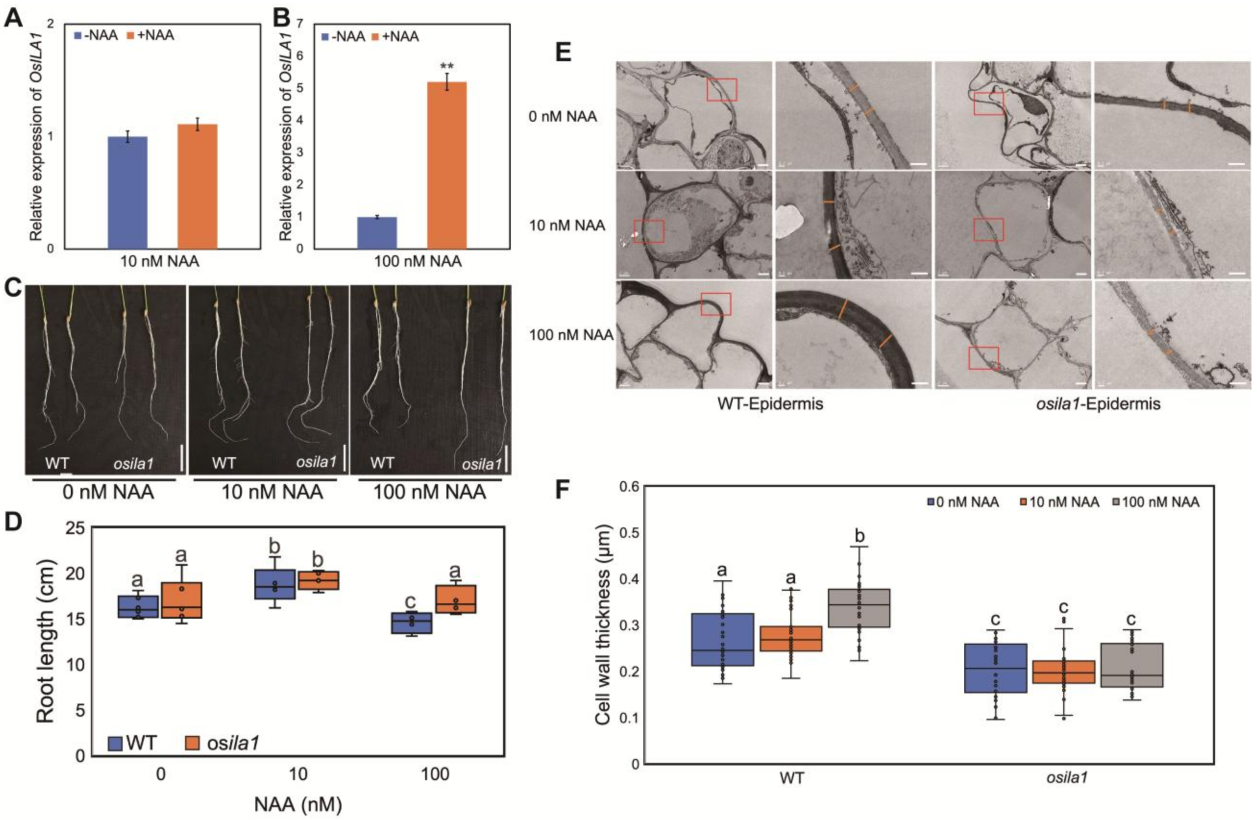

Fig. S5. OsILA1 functions in high-level auxin signaling. (**A**) Relative expression changes of *OsILA1* under 10 nM NAA treatment for 2 hrs. Error bars are ± SE, n = 3. (**B**) Relative expression changes of *OsILA1* under 100 nM NAA treatment for 2 hrs. Error bars are ± SE, n = 3. Student's *t*-test: $^{**}P < 0.01$. (**C**) Representative root images of root systems of WT and *osila1* under 0 nM, 10 nM, and 100 nM NAA treatments. Scale bars, 1 cm. (**D**) Root length analysis of WT and *osila1* after 0 nM, 10 nM, and 100 nM NAA treatments. Error bars are ± SD, n = 12. Different letters indicate significant differences, $P < 0.01$ from a one-way ANOVA. (**E**) Representative cell wall images of the WT and *osila1* root epidermis under 0 nM, 10 nM, and 100 nM NAA treatments for 3 hrs. The left pictures of WT and *osila1* are epidermis cells. Scale bars, 2 μm. The magnified view on the right is the region marked by the red box. The orange line indicates the cell wall. Scale bars, 0.5 μm. (**F**) The cell wall thickness of the WT and *osila1* root epidermis under 0 nM, 10 nM, and 100 nM NAA treatments. Error bars are ± SD, n ≥ 30 positions. Different letters indicate significant differences, $P < 0.01$ from using a one-way ANOVA.

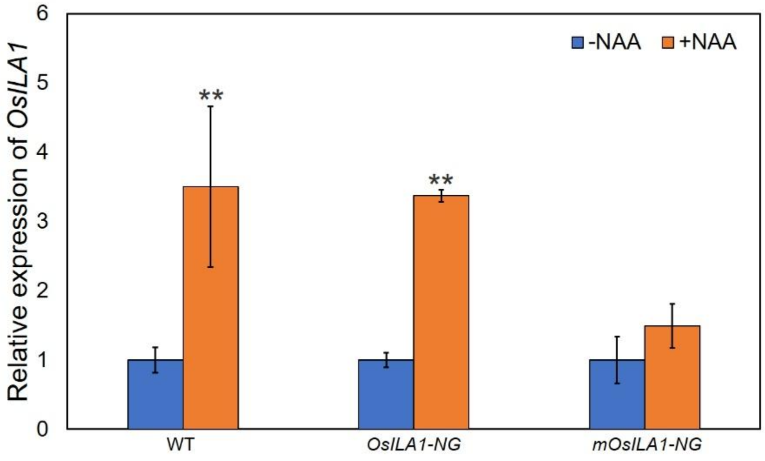

Fig. S6. Relative expression changes of *OsILA1* in WT, *OsILA1-NG*, and *mOsILA1-NG* lines with/without 100 nM NAA treatment for 3 hrs. Error bars are ± SE, n = 3. Student's *t*-test: $**P < 0.01$.

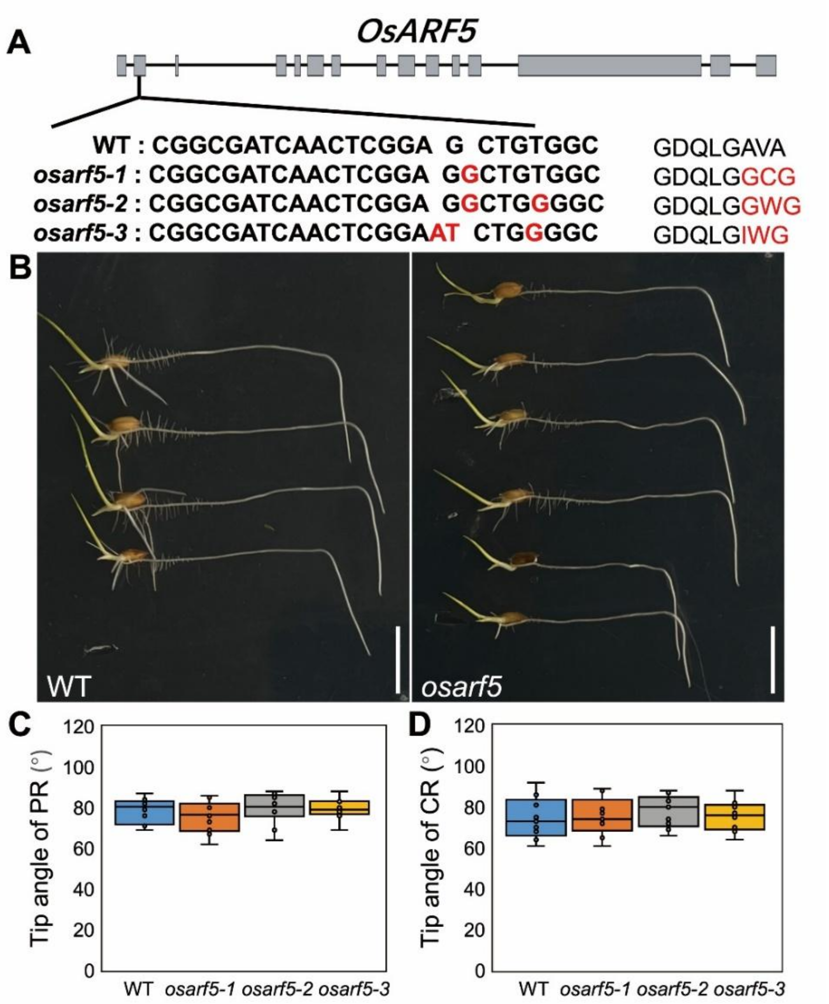

Fig. S7. *OsARF5* mutants exhibit normal gravitropism. (**A**) Knockout mutants of *OsARF5* via CRISPR/Cas9. (**B**) Representative root images of gravitropic responses in WT and *osarf5*/*osarf5-1*. Scale bars, 1 cm. (**C**) Tip angle analysis of WT and *osarf5* PR after 12-h gravitropic responses. Error bars are ± SD, n = 15. Student's *t*-test: $^{**}P < 0.01$. (**D**) Tip angle analysis of WT and *osarf5* CR after 12-h gravitropic responses. Error bars are ± SD, n = 20. Student's *t*-test: $^{**}P < 0.01$.

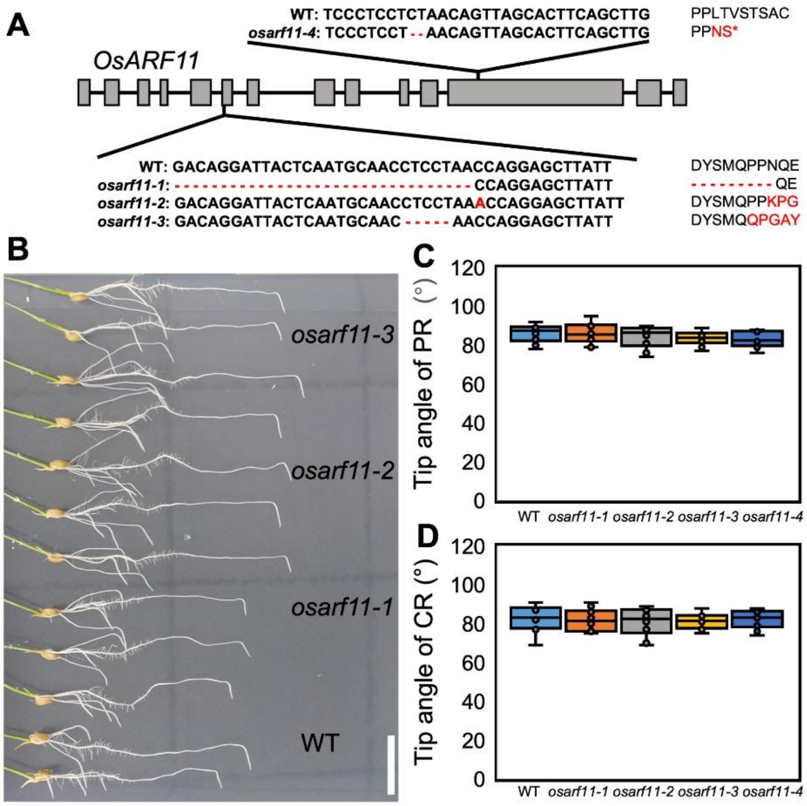

Fig. S8. ***OsARF11* mutants exhibit normal gravitropism.** (**A**) Knockout mutants of *OsARF11* via CRISPR/Cas9. (**B**) Representative root images of gravitropic responses in WT and *osarf11*/*osarf11-1*. Scale bars, 1 cm. (**C**) Tip angle analysis of WT and *osarf11* PR after 12-h gravitropic responses. Error bars are ± SD, n = 12. (**D**) Tip angle analysis of WT and *osarf11* CR after 12-h gravitropic responses. Error bars are ± SD, n = 22.

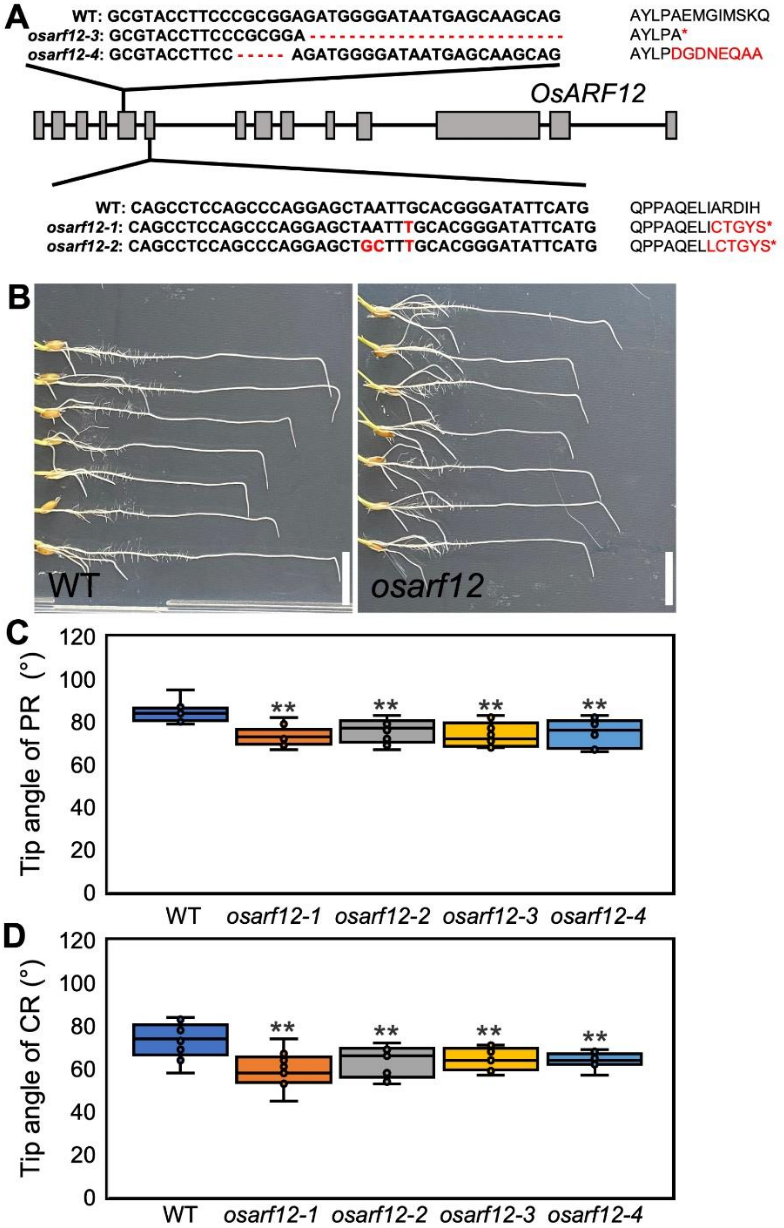

Fig. S9. *OsARF12* mutants exhibit reduced gravitropism. (**A**) Knockout mutants of *OsARF12* via CRISPR/Cas9. (**B**) Representative root images of gravitropic responses in WT and *osarf12*/*osarf12-1*. Scale bars, 1 cm. (**C**) Tip angle analysis of WT and *osarf12* PR after 12-h gravitropic responses. Error bars are ± SD, n = 15. Student's *t*-test: $^{**}P < 0.01$. (**D**) Tip angle analysis of WT and *osarf12* CR after 12-h gravitropic responses. Error bars are ± SD, n = 25. Student's *t*-test: $^{**}P < 0.01$.

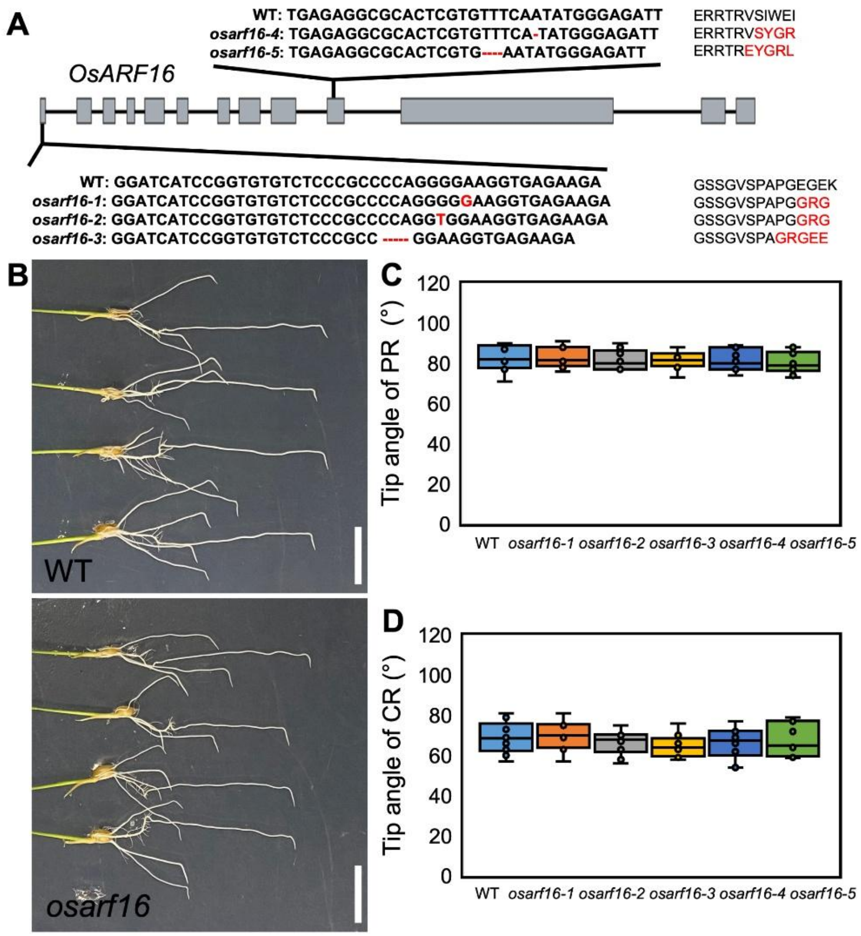

Fig. S10. ***OsARF16* mutants exhibit normal gravitropism.** (**A**) Knockout mutants of *OsARF16* via CRISPR/Cas9. (**B**) Representative root images of gravitropic responses in WT and *osarf16*/*osarf16-1*. Scale bars, 1 cm. (**C**) Tip angle analysis of WT and *osarf16* PR after 12-h gravitropic responses. Error bars are ± SD, n = 14. (**D**) Tip angle analysis of WT and *osarf16* CR after 12-h gravitropic responses. Error bars are ± SD, n = 22.

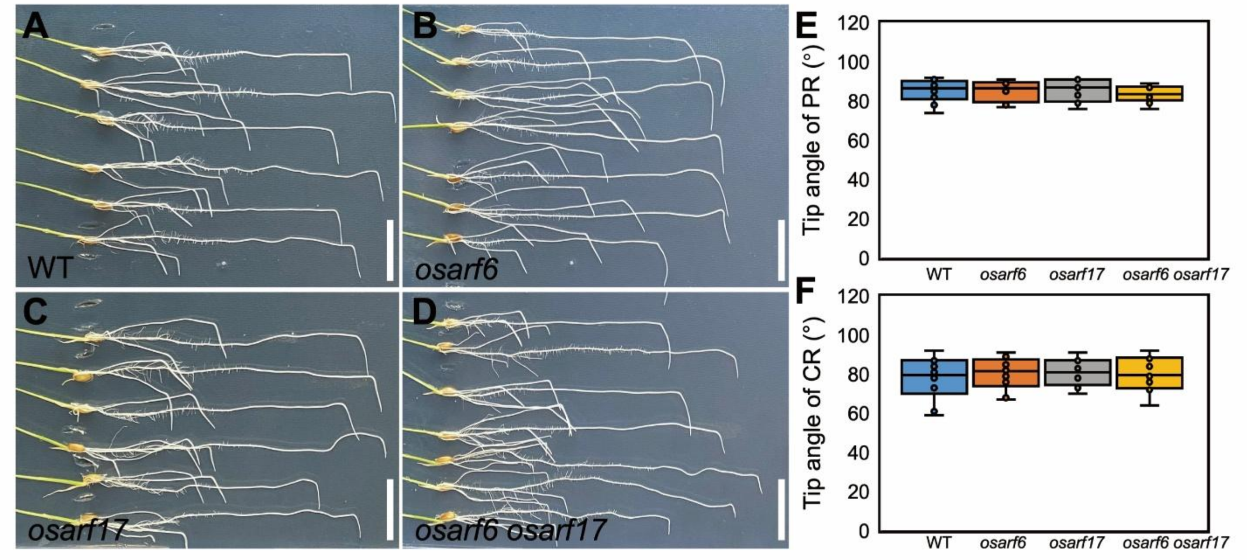

Fig. S11. *OsARF6* and *OsARF17* mutants exhibit normal gravitropism. (**A**-**D**) Representative root images of gravitropic responses in WT, *osarf6*, *osarf17*, and *osarf6 osarf17*. Scale bars, 1 cm. (**E**) Tip angle analysis of WT, *osarf6*, *osarf17*, and *osarf6 osarf17* PR after 12-h gravitropic responses. Error bars are ± SD, n = 10. (**F**) Tip angle analysis of WT, *osarf6*, *osarf17* and *osarf6 osarf17* CR after 12-h gravitropic responses. Error bars are ± SD, n = 20.

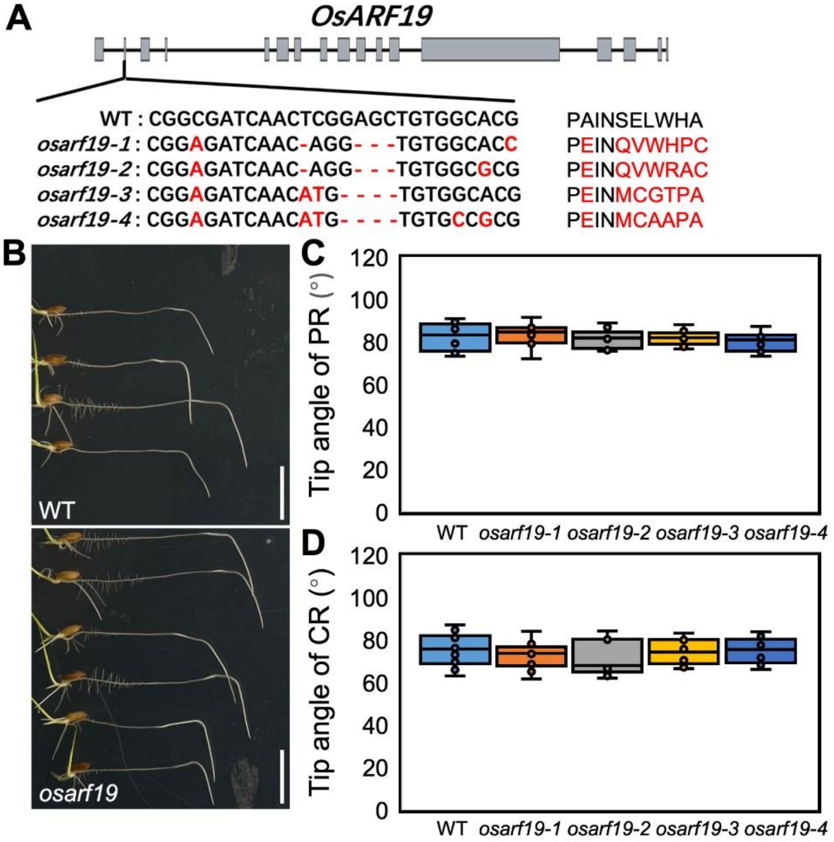

Fig. S12. *OsARF19* mutants exhibit normal gravitropism. (**A**) Knockout mutants of *OsARF19* via CRISPR/Cas9. (**B**) Representative root images of gravitropic responses in WT and *osarf19*/*osarf19-1*. Scale bars, 1 cm. (**C**) Tip angle analysis of WT and *osarf19* PR after 12-h gravitropic responses. Error bars are ± SD, n = 14. (**D**) Tip angle analysis of WT and *osarf19* CR after 12-h gravitropic responses. Error bars are ± SD, n = 22.

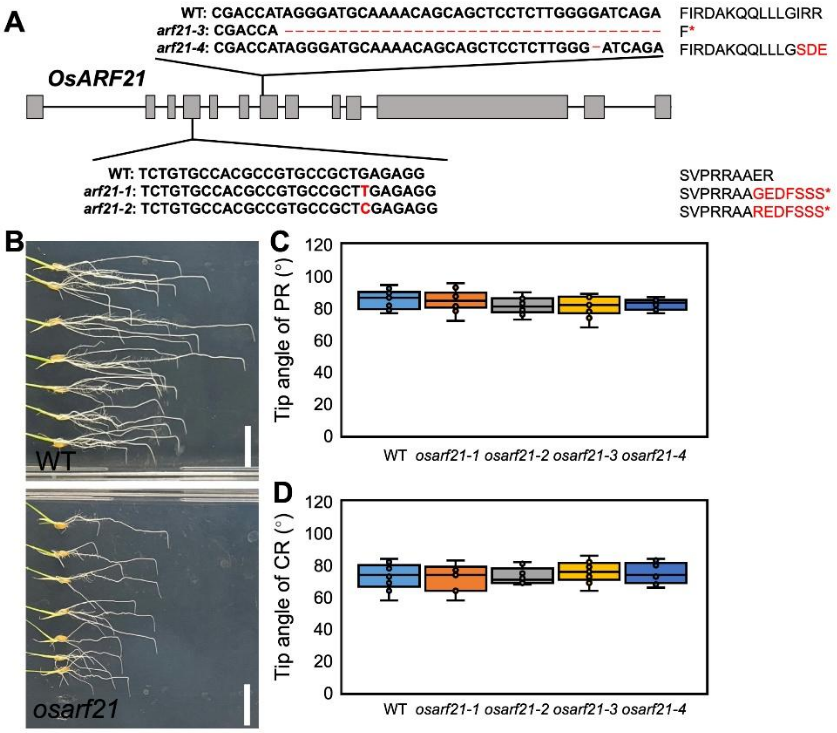

Fig. S13. *OsARF21* mutants exhibit normal gravitropism. (**A**) Knockout mutants of *OsARF21* via CRISPR/Cas9. (**B**) Representative root images of gravitropic responses in WT and *osarf21*/*osarf21-1*. Scale bars, 1 cm. (**C**) Tip angle analysis of WT and *osarf21* PR after 12-h gravitropic responses. Error bars are ± SD, n = 12. (D) Tip angle analysis of WT and *osarf21* CR after 12-h gravitropic responses. Error bars are ± SD, n = 24.

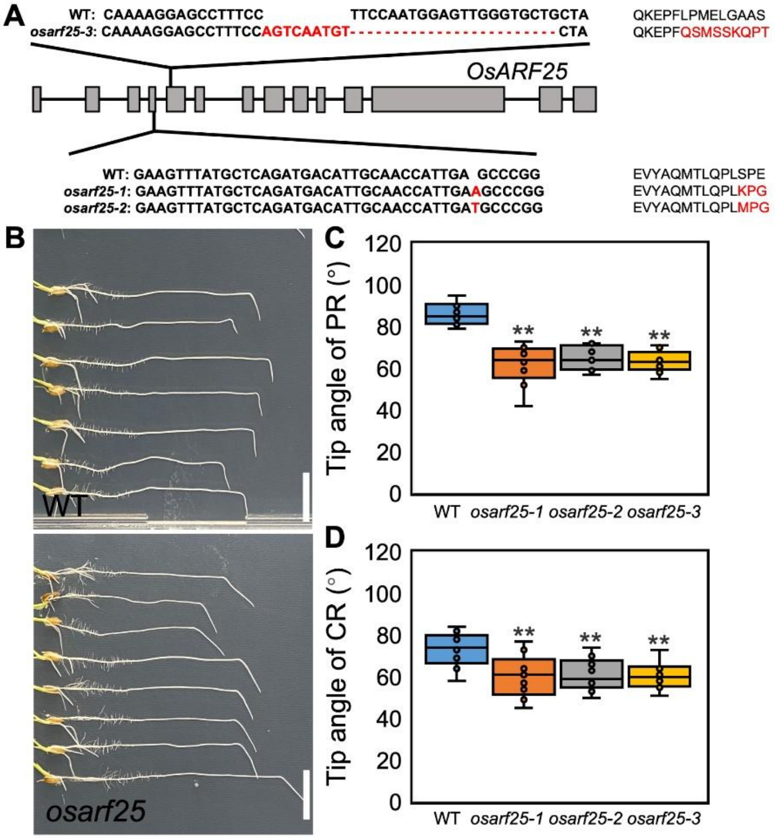

Fig. S14. *OsARF25* mutants exhibit reduced gravitropism. (**A**) Knockout mutants of *OsARF25* via CRISPR/Cas9. (**B**) Representative root images of gravitropic responses in WT and *osarf25*/*osarf25-1*. Scale bars, 1 cm. (**C**) Tip angle analysis of WT and *osarf25* PR after 12-h gravitropic responses. Error bars are ± SD, n = 15. Student's *t*-test: $^{**}P < 0.01$. (**D**) Tip angle analysis of WT and *osarf25* CR after 12-h gravitropic responses. Error bars are ± SD, n = 25. Student's *t*-test: $^{**}P < 0.01$.

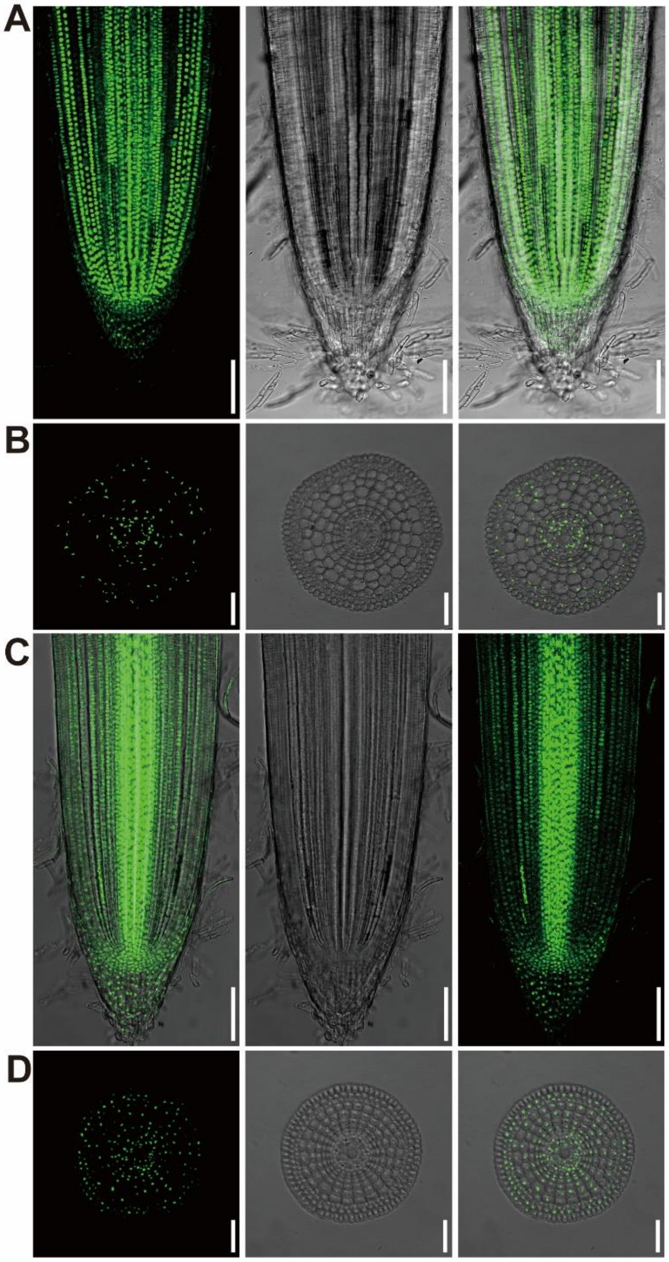

Fig. S15. Expression pattern of *OsARF12* and *OsARF25*. (**A**) Representative confocal images, bright field images and merge images of the root longitudinal section of rice *proOsARF12::VENUS-N7* transcriptional reporter. Scale bar, 100 μm. (**B**) Representative confocal images, bright field images and merge images of the root transverse section of rice *proOsARF12::VENUS-N7* transcriptional reporter. Scale bar, 100 μm. (**C**) Representative confocal images, bright field images and merge images of the root longitudinal section of rice *proOsARF25::VENUS-N7* transcriptional reporter. Scale bar, 100 μm. (**D**) Representative confocal images, bright field images and merge images of the root transverse section of rice *proOsARF25::VENUS-N7* transcriptional reporter. Scale bar, 100 μm.

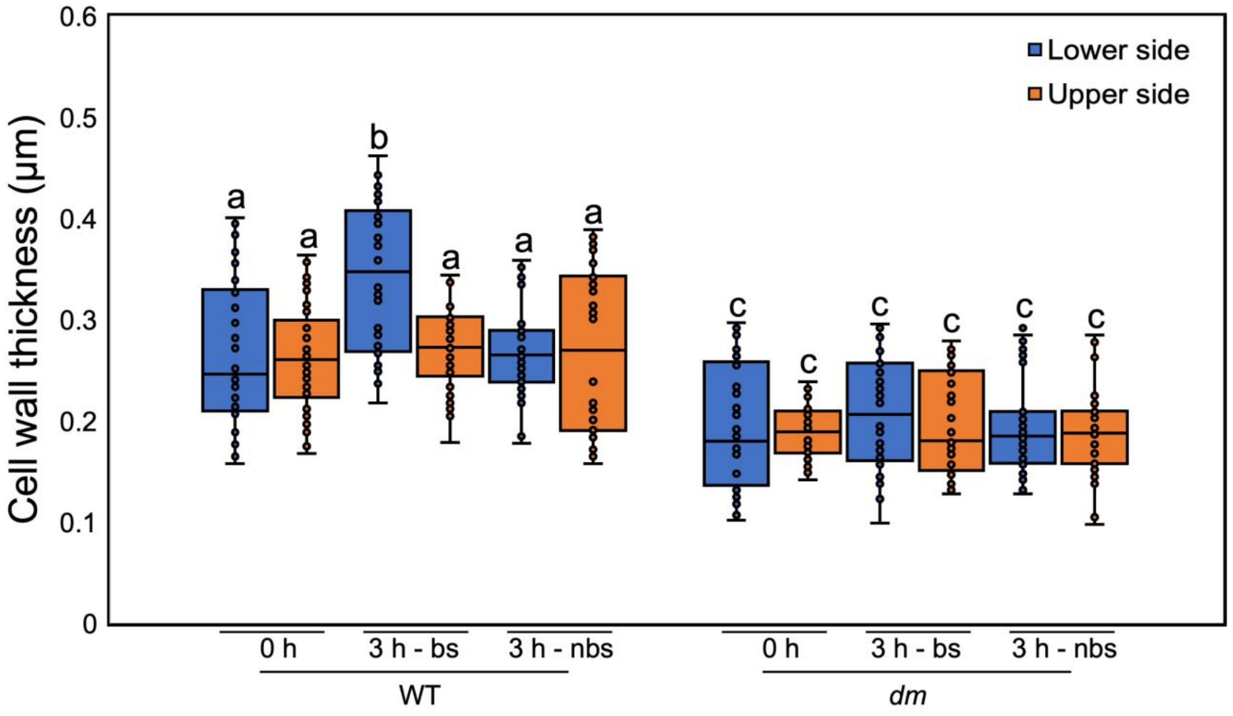

Fig. S16. The cell wall thickness of the upper and lower sides of the primary root (PR) epidermis at 0 and after 3-h of gravistimulation. Error bars are ± SD, n ≥ 30 positions. Different letters indicate significant differences, $P < 0.01$ from using a one-way ANOVA.

Table S1. List of primers used in this work.

Description	Forward primer (5'→3')	Reverse primer (5'→3')
osarf5 mutant identification	ATTGAACCGGTTGCAGCTCC	CAGCCGCTGGCCATTAATCC
osarf11 mutant identification	TTCAGGTGCCAAGAAGGTGA	TATTCAGTGGGGTGCTTGCTT
osarf12 mutant identification	AAGGTGGGTGGCTAGGTTTG	CCTGCTGCACGAATGAACAC
osarf16 mutant identification	AGTGCGCTGGAAGAACTCTC	AATGCGTATGCACGTTGGTG
osarf19 mutant identification	ACGAGTAGCTGCTACTGCAGT	GCATTCCACCTCTCAAGAACTCAT
osarf21 mutant identification	CCAGGTCAGCCTAAAAGGCA	GTTATTCGCTGCAGCATGGG
osarf25 mutant identification	TTGGCTCTGTTCATTGCCCT	TCAACAAAATCCGGCCATGC
OsARF12 promoter	ACATGATTACGAATTCCGGTACAGCGATCGAACAGG	TGCTCACCATGAGCTCCTCCTCCTCCTCCGACCAAAC
OsARF25 promoter	GATTACGAATTCGAGAAGCAAAGTACCCCTTGGCC	CTTGCTCACCATGAGCTCCAATCACCAGTGCACCC
OsILA1 promoter	CATGATTACGAATTCGAGCTCCGGGTCCATTCTTAGGCT	GGATACATGGGTACCGAGCGCCGACCTGTTCGACG
OsILA1 cDNA	GTTCCAGATTACGCTCCCATGGACCACGGCGGTCA	CGACTCTAGAGGATCCCCTCAATGGACCTTCCCAAAAGTAAAGAAGC
AD-OsARF12	CAGTGAATTCCACCCAATGAGCTCGTCGTCGGC	TATCGATGCCCACCCTCAGGACAGATACCGTGGATCAT
AD-OsARF25	CAGTGAATTCCACCCAATGAAGCTCTCTCCGCCG	TATCGATGCCCACCCTCAGTAGTCCAGAGGCGCTACAG
OsILA1 qRT-PCR	GCGGAAGGTCAGGAGAGAAC	TCCATGGCATAACTGGCGTT
CF *OsILA1* promoter	TTCCAGACTCCATCCACCCA	GGTAGAGAAAAACAGGAGAGGC
CF1 *OsILA1* promoter	CTCGTCATCGGTGGACAAATT	CAAACAGCAAAGGCGCAA
CF2 *OsILA1* promoter	TTGCGCCTTTGCTGTTTG	CCCAGCCTCATGTGTTCTC
